# Identification and Characterization of Long Non-coding RNAs in the Intestine of Olive Flounder (*Paralichthys olivaceus*) During *Edwardsiella tarda* Infection

**DOI:** 10.3389/fimmu.2021.623764

**Published:** 2021-03-31

**Authors:** Yunji Xiu, Yingrui Li, Xiaofei Liu, Lin Su, Shun Zhou, Chao Li

**Affiliations:** ^1^School of Marine Science and Engineering, Qingdao Agricultural University, Qingdao, China; ^2^College of Marine Science and Engineering, Nanjing Normal University, Nanjing, China

**Keywords:** long non-coding RNA, *Paralichthys olivaceus*, *Edwardsiella tarda*, intestinal mucosal immune response, lncRNA-miRNA-mRNA

## Abstract

Long non-coding RNAs (lncRNAs) play widespread roles in fundamental biological processes, including immune responses. The olive flounder (*Paralichthys olivaceus*), an important economical flatfish widely cultured in Japan, Korea, and China, is threatened by infectious pathogens, including bacteria, viruses, and parasites. However, the role of lncRNAs in the immune responses of this species against pathogen infections is not well-understood. Therefore, in this study, we aimed to identify lncRNAs in the intestine of olive flounder and evaluate their differential expression profiles during *Edwardsiella tarda* infection, which is an important zoonotic and intestinal pathogen. A total of 4,445 putative lncRNAs were identified, including 3,975 novel lncRNAs and 470 annotated lncRNAs. These lncRNAs had shorter lengths and fewer exons compared with mRNAs. In total, 115 differentially expressed lncRNAs (DE-lncRNAs) were identified during *E. tarda* infection. To validate the expression pattern of lncRNAs, six DE-lncRNAs were randomly selected for quantitative real-time PCR. The co-located and co-expressed mRNAs of DE-lncRNAs were predicted, which were used to conduct the Gene Ontology (GO) and Kyoto Encyclopedia of Genes and Genomes (KEGG) enrichment analyses. The target genes of DE-lncRNAs enriched numerous immune-related processes and exhibited a strong correlation with immune-related signaling pathways. To better understand the extensive regulatory functions of lncRNAs, the lncRNA–miRNA–mRNA regulatory networks were constructed, and two potential competing endogenous RNA (ceRNA) networks, LNC_001979-novel_171-Potusc2 and LNC_001979-novel_171-Podad1, were preliminarily identified from the intestine of olive flounders for the first time. In conclusion, this study provides an invaluable annotation and expression profile of lncRNAs in the intestine of olive flounder infected with *E. tarda*; this forms a basis for further studies on the regulatory function of lncRNAs in the intestinal mucosal immune responses of olive flounder.

## Introduction

Long non-coding RNAs (lncRNAs) are a group of non-coding RNAs (ncRNAs) that are longer than 200 nucleotides (1). Although a few lncRNAs have been reported to encode small peptides (2, 3), most lncRNAs cannot translate into proteins. Compared with coding genes, lncRNAs have fewer and longer exons, and exhibit more tissue-specific expression and lower expression (1). LncRNAs have been divided into different categories based on their length, transcript properties, genomic location, regulatory elements, and function, in which three classes of lncRNAs (intergenic lncRNAs, antisense lncRNAs, and intronic lncRNAs) are known based on their genomic location (4). Lacking sequence conservation across different species, lncRNAs were initially considered as transcriptional noise and their biological importance was doubted (1). However, studies have shown that lncRNAs play important roles in the regulation of immune responses and host defense against pathogens (5). Several lncRNAs have been shown to be differentially expressed during microbial component stimulation or pathogen infection (5). Upon microbial component stimulation, the lncRNAs might regulate the transcription of immune genes by interacting with other complexes. In addition, these lncRNAs might play an important role in controlling host–pathogen interactions via regulating the growth and replication of pathogens, or via cell-autonomous anti-microbial defense mechanisms. In conclusion, lncRNAs can regulate a variety of biological processes at transcriptional and post-transcriptional levels, such as DNA methylation, histone modification, splicing, transcription, and translation, by interacting with genomic DNA, RNA, proteins, or a combination of these (1). Recently, several novel findings suggested that lncRNAs can act as miRNA sponges to bind miRNAs competitively to modulate the expression of mRNAs.

LncRNAs have been extensively studied in teleosts and emerging evidence suggests that lncRNAs may also serve as important regulators in the immune responses of teleosts (2). In teleosts, a number of lncRNAs have been shown to be differentially expressed during pathogen infections. Through comparative transcriptome data analysis, lncRNAs from rainbow trout (*Oncorhynchus mykiss*) (6), Atlantic salmon (*Salmo salar*) (7–10), Coho salmon (*Oncorhynchus kisutch*) (10), large yellow croakers (*Larimichthys crocea*) (11), zebrafish (*Danio rerio*) (12), European sea bass (*Dicentrarchus labrax*) (13), and Nile tilapia (*Oreochromis niloticus*) (3) were widely modulated after viruses, bacteria, or parasite infections. Enrichment analysis revealed that the modulated lncRNAs were localized near immune- and stress- related genes (10, 13). Previous studies have confirmed that lncRNAs might be implicated in teleost immune responses to pathogen infections. However, further analyses are required to fully characterize their detailed functions and mechanisms.

Despite the evidence for the immune-related regulatory functions of lncRNAs, few studies have been conducted on olive flounder (*Paralichthys olivaceus*). Olive flounder, which is an important economical flatfish, has been widely cultured in Japan, Korea, and China (14). However, the olive flounder industry is threatened by infectious pathogens, including bacteria, viruses, and parasites (15), which cause mixed infectious diseases, numerous deaths, and huge economic losses (16). Acting as a critical zoonotic and intestinal pathogen, *Edwardsiella tarda* could also result in substantial economic losses to the olive flounder aquaculture industry (16). A previous study identified 10,270 lncRNAs from mixed immune-related tissues (gill, intestine, liver, and kidney) in olive flounder with the PacBio Sequel platform, which consists of 38.18% antisense, 32.62% sense intronic, 20.58% lincRNA, and 8.62% sense overlapping lncRNA (17). In addition, the expression pattern and function of lncRNAs in the skeletal muscle of olive flounder have been characterized, which indicated that lncRNAs may participate in the development of skeletal muscle through cis- or trans-acting mechanisms (18). In summary, previous studies have provided a scientific basis for further studies on the biological function of lncRNAs in olive flounder, and these lncRNAs are greatly in need of further investigation. Considering that lncRNAs play important roles in modulating the immune responses of teleosts, it is necessary to further characterize the regulatory function and mechanism of lncRNAs in olive flounder.

In this study, lncRNAs were identified and characterized from the intestine of olive flounder. Besides serving as the prime site for nutrient absorption, the intestine represent one of the first lines of defense (19). Moreover, the expression patterns of lncRNAs at different time points post *E. tarda* infection were characterized. Additionally, co-localization and co-expression analyses were performed to predict the potential lncRNA–mRNA interactions in response to bacterial infections. GO and KEGG enrichment analyses were carried out with the targeted mRNA of lncRNAs. Moreover, the competing endogenous RNA (ceRNA) network, lncRNA–miRNA–mRNA, was constructed with differentially expressed lncRNAs, miRNAs, and mRNAs that had been reported previously (14). In conclusion, this study provides the expression and function analysis of newly identified lncRNAs from the intestine of olive flounder, which is one of the main mucosa-associated lymphoid tissues of teleosts. Our study provides insights into intestinal immune responses of lncRNAs during host–pathogen interactions and lays the foundation for further functional studies on lncRNAs during pathogen infections.

## Methods and Materials

### Experimental Fish, Bacteria Challenge, and Sample Collection

The experimental fish, bacteria challenge, and sample collection have been described in a previous study (14). Briefly, a total of 50 olive flounders (body weight 120 ± 10 g, body length 22 ± 3 cm) were purchased from Huanghai Aquaculture Company, Shandong, China and raised at 20 ± 1°C in a recirculating water system for 1 week before the experiments, during which they were fed twice a day with a commercial diet. The health of the experimental fish was confirmed by randomly sampling for bacteriological, parasitological, and virological examinations. In the challenge experiment, a total of 27 olive flounders were immersed in the *E. tarda* solution with a final concentration of 6 × 10^7^ CFU/ml for 2 h and then returned to the recirculating water system. Then, the posterior intestine from nine fish were collected at 2, 8, and 12 h post-immersion, which was designated as H2, H8, and H12, respectively. In the control group, the *E. tarda* solution was replaced with aseptic seawater, and the posterior intestine from nine fish were collected and designated as H0. Overall, this experiment included four time points (H0, H2, H8, and H12), and each time point contained three biological replicates that consisted of three fish for each one. The posterior intestine was quickly isolated and frozen immediately in liquid nitrogen until RNA isolation.

### RNA Isolation and Library Preparation for lncRNA Sequencing

Total RNA from the posterior intestine was isolated using the Trizol reagent (Invitrogen, USA) following the manufacturer's protocol. RNA degradation and contamination were assessed on a 1% agarose gel. RNA purity was monitored using the NanoPhotometer® spectrophotometer (IMPLEN, CA, USA). RNA concentration was assessed using the RNA Assay Kit in Qubit® 2.0 Flurometer (Life Technologies, CA, USA). RNA integrity was measured using the RNA Nano 6000 Assay Kit of the Bioanalyzer 2100 system (Agilent Technologies, CA, USA). The library sequencing was performed by Novogene Corporation (Tianjin, China). A total amount of 3 μg of RNA per sample was used as the input material for the RNA sample preparations and sequencing. First, ribosomal RNA (rRNA) was removed using the Ribo-zero™ rRNA Removal Kit (Epicentre, USA), and rRNA-free residue was cleaned up by ethanol precipitation. Subsequently, sequencing libraries were generated using the rRNA-depleted RNA using the Ultra™ Directional RNA Library Prep Kit for Illumina® (NEB, USA) following the manufacturer's instructions. Briefly, fragmentation was conducted using divalent cations under elevated temperatures in the NEBNext First Strand Synthesis Reaction Buffer (5X). The first strand of cDNA was synthesized using a random hexamer primer and M-MuLV Reverse Transcriptase (RNaseH-), while the second strand of cDNA was synthesized using DNA Polymerase I and RNase H. In order to select cDNA fragments that were ~150–200 bp in length, the library fragments were purified with the AMPure XP system (Beckman Coulter, Beverly, USA). Then, 3 μL of USER Enzyme (NEB, USA) was used with size-selected, adaptor-ligated cDNA at 37°C for 15 min followed by 5 min at 95°C before PCR. Thereafter, PCR was performed with Phusion High-Fidelity DNA polymerase, universal PCR primers, and index (X) primers. Next, the products were purified (AMPure XP system) and their library quality was assessed on the Agilent Bioanalyzer 2100 system. Following the manufacturer's instructions, the clustering of the index-coded samples was then performed on a cBot Cluster Generation System using TruSeq PE Cluster Kit v3-cBot-HS (Illumia). Finally, after the cluster generation, the libraries were sequenced on an Illumina Hiseq 4000 platform and 150 bp paired-end reads were generated.

### Transcriptome Assembly and lncRNA Identification

Raw reads were first processed through in-house Perl scripts, in which clean reads were acquired by removing low-quality reads that contained adapter sequences and ploy-N from the raw data. At the same time, the Q20, Q30, and GC content of the clean data were calculated. The high-quality clean data were used for the subsequent downstream analyses. Then, the reference genome and gene model annotation files were downloaded from the genome website directly (ftp://ftp.ncbi.nlm.nih.gov/genomes/all/GCF/001/970/005/GCF_001970005.1_Flounder_ref_guided_V1.0/). The reference genome index was built using bowtie2 v2.2.8 and paired-end clean reads were aligned to the reference genome using HISAT2 v2.0.4 (20). Next, the mapped reads of each sample were assembled using StringTie v1.3.1 (21) in a reference-based approach. StringTie uses a novel network flow algorithm as well as an optional *de novo* assembly step to assemble and quantitate full-length transcripts that represent multiple splice variants for each gene locus.

Based on the transcriptase splicing results, we set a series of strict screening conditions based on the structural characteristics of lncRNAs and the functional characteristics of non-coding proteins. We followed the five basic principles below to filter lncRNAs: (1) exon number ≥ 2; (2) transcript length > 200 bp; (3) filter annotated transcripts and lncRNAs; (4) expression level (Cuffquant software, FPKM ≥ 0.5); (5) transcripts with coding potential predicted by CNCI (Coding-Non-Coding-Index), CPC (Coding Potential Calculator), Pfam Scan, and PhyloCSF (phylogenetic codon substitution frequency) were filtered out, and those without coding potential were our candidate set of lncRNAs.

### Different Expression Analysis of lncRNAs and qRT-PCR Verification

Gene expression was normalized using the fragments per kilobase of exon per million reads mapped (FPKM), which was calculated using Cuffdiff (v2.1.1) (22). Cuffdiff provides statistical routines for determining differential expression in the gene expression data using a model based on the contrary binomial distribution (22). Subsequently, differentially expressed lncRNAs at H2, H8, and H12 compared with H0 were filtered, and lncRNAs with a *p*-value < 0.05 were assigned as differentially expressed.

To validate the Illumina sequencing data, a total of six differentially expressed lncRNA were randomly selected for the qRT-PCR analysis. First, cDNA was synthetized using the PrimeScript 1st strand cDNA Synthesis Kit (Takara, Japan). Then, specific primers were designed based on their sequences and EF1α was used as the internal control. The qRT-PCR was performed with the CFX96 Real-time Fluorescent quantitative PCR system (Bio-Rad, USA) using TB Green^TM^ Premix Ex Taq^TM^ II (TaKaRa, Japan). The amplification cycle was as follows: 95°C for 30 s, 40 cycles at 95°C for 5 s, and 60°C for 1 min, followed by a melting curve from 60 to 95°C. Data are shown as means ± SE for three replicates, and statistical analysis was performed using SPSS19.0.

### Target Gene Prediction and Enrichment Analysis

The target genes of the DE-lncRNAs were predicted using *cis*/*trans*-regulatory algorithms. The *cis* and *trans* regulatory roles refer to the influence of lncRNAs on neighboring target genes and other genes at the expression level, respectively. We searched coding genes that were 10/100 k upstream and downstream of lncRNA, which were considered to be co-located target genes. Co-expressed target genes were predicted using the expressed Pearson correlation coefficient between the lncRNAs and corresponding coding genes using custom scripts (*p* < 0.05 and |R| > 0.95). Finally, regulatory networks were constructed and visualized by Cytoscape v3.6.1. In this case, the coding genes used for target gene prediction have been previously reported based on the immune responses of *P. olivaceus* against an *E. tarda* challenge (14).

The Gene Ontology (GO) enrichment analysis of lncRNA target genes was performed using the GOseq package in R, in which gene length bias was corrected (23). GO terms with *p* < 0.05 were considered significantly enriched by differentially expressed genes. KEGG is a database that contains large-scale molecular datasets generated by genome sequencing and other high-throughput experimental technologies (http://www.genome.jp/kegg/) that can be used to elucidate the high-level functions and utilities of biological systems (24), such as cells, organisms, and ecosystems, from molecular-level information. We used the KOBAS software to test the statistical enrichment of the lncRNA target genes in KEGG pathways (25).

### Construction of the lncRNA–miRNA–mRNA Regulatory Network

To better understand the extensive regulatory functions of lncRNAs, the lncRNA mediated ceRNA network was constructed with differentially expressed miRNAs and mRNAs that have been reported previously (14). The lncRNA–miRNA and miRNA–mRNA interaction analysis was conducted with the microRNA target prediction tool miRanda, and the lncRNA–miRNA–mRNA network was generated using a combination of the lncRNA–miRNA network and miRNA–mRNA network with the Cytoscape 3.6.1 software.

### Luciferase Assay

To test the interaction between LNC_001979 or mRNA (Potusc2, Podad1) and novel_171, luciferase reporter assays were conducted using the dual-luciferase reporter system. The wild-type target sequences of LNC_001979, Potusc2, and Podad1 were cloned into the pmirGLO reporter luciferase vector and named pmirGLO-LNC_001979-WT, pmirGLO-Potusc2-WT, and pmirGLO-Podad1-WT, respectively. Then, the wild-type recombinant plasmids were mutated into pmirGLO-LNC_001979-Mut, pmirGLO-Potusc2-Mut, and pmirGLO-Podad1-Mut with mutant primers and the In-fusion HD Cloning Kit (Takara, Japan) following the manufacturer's instructions. HEK293T cells were co-transfected with wild-type or mutant-type recombinant plasmid and novel_171 mimics or negative control mimics (NC) using Lipofectamine 2000 (Invitrogen, USA). The cells were collected at 48 h after transfection and the luciferase activity was detected using the Luciferase Assay Systems kit (Promega, USA) following the manufacturer's instructions.

## Results

### Genome-Wide Identification of lncRNAs

**Figure 1:**
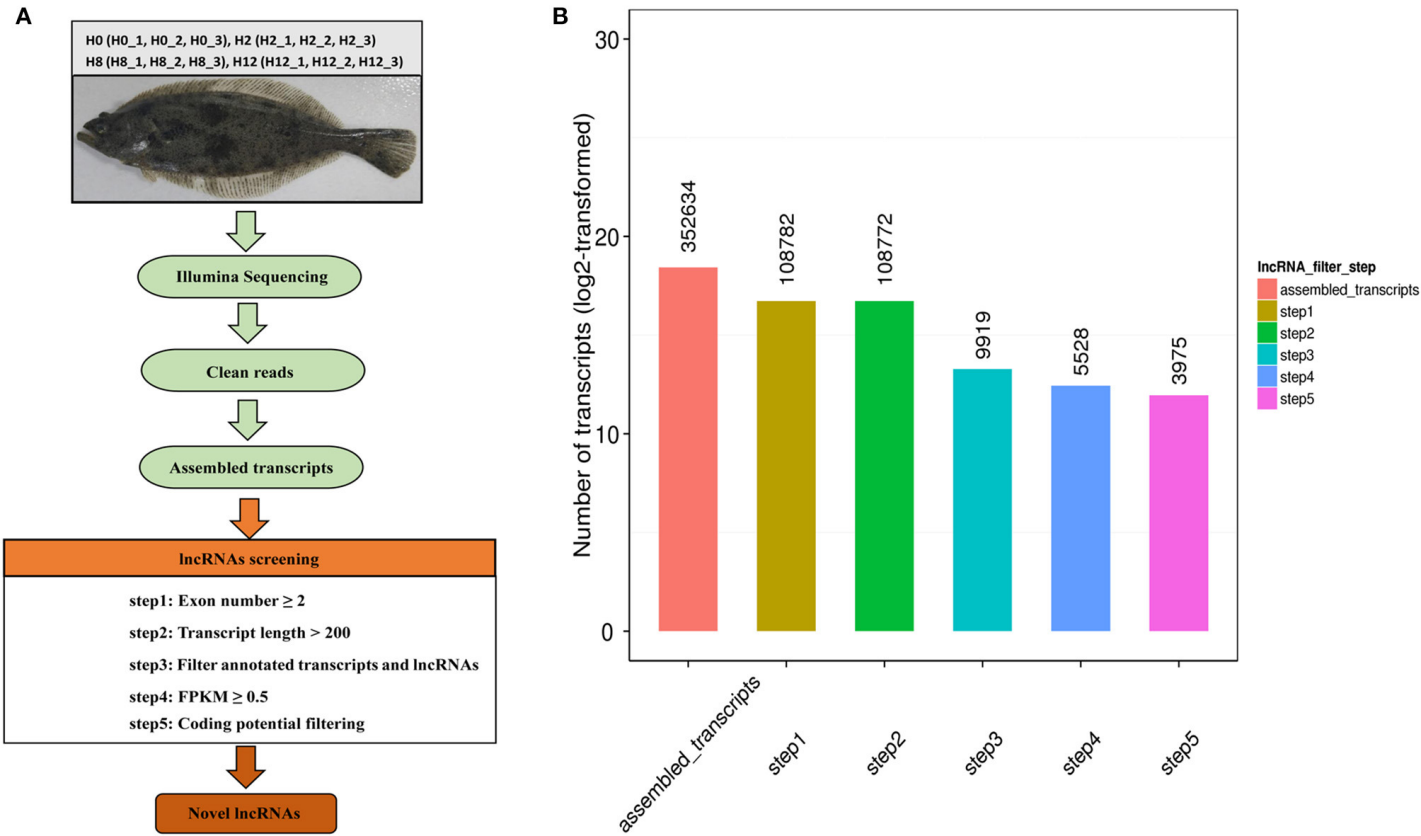
**(A)** Identification pipeline for lncRNAs in olive flounders. **(B)** The numbers of candidate transcripts at five filtering stages.

Twelve cDNA libraries were constructed to perform Illumina sequencing. The data has been deposited in NCBI database, with the BioProject number of PRJNA510440. The raw reads, clean reads, clean bases, error rate, Q20, Q30, and GC contents for each library are shown in Table 1. A total of 305,559,108, 308,953,538, 259,257,204, and 335,588,542 raw reads were acquired from the H0, H2, H8, and H12 group, respectively. All the libraries were of good quality, with clean base values ≥12.25 G, error rates ≤ 0.02, Q20 ≥ 96.99%, and Q30 ≥ 92.32%. Therefore, all the libraries were verified in be appropriate for further study. Then, the clean reads from all the libraries were used to discern the lncRNAs. As shown in Figure 1A, the lncRNAs were obtained following the five basic filtering steps, and a total of 108,782, 108,772, 9,919, 5,528, and 3,975 transcripts were identified from step 1 to step 5, respectively (Figure 1B). Finally, a total of 4,445 putative lncRNAs were identified, including 3,975 novel lncRNAs and 470 annotated lncRNAs (Supplementary Table 1).

**Table 1:** Information of lncRNAs sequencing data.

**Sample Name**	**Raw Reads**	**Clean Reads**	**Clean Bases**	**Error Rate**	**Q20 (%)**	**Q30 (%)**	**GC Content**
HO_1	91,001,552	85,570,680	12.84 G	0.02	97.16	92.72	47.40
HO_2	98,086,488	92,224,408	13.83 G	0.02	97.18	92.72	48.90
HO_3	116,471,068	109,413,634	16.41 G	0.02	96.99	92.32	48.85
H2_1	133,971,546	125,928,236	18.89 G	0.02	97.10	92.55	49.09
H2_2	91,713,816	86,756,860	13.01 G	0.02	97.19	92.72	49.33
H2_3	83,268,176	81,661,360	12.25 G	0.02	97.29	92.87	49.59
H8_1	84,134,060	82,485,038	12.37 G	0.02	97.33	92.97	49.47
H8_2	89,841,602	88,149,192	13.22 G	0.02	97.33	92.98	49.26
H8_3	85,281,542	83,698,290	12.55 G	0.01	97.39	93.10	49.37
H12_1	123,787,190	121,500,194	18.23 G	0.01	97.39	93.09	49.28
H12_2	99,739,544	97,782,904	14.67 G	0.02	97.33	92.96	49.00
H12_3	112,061,808	109,856,848	16.48 G	0.01	97.37	93.09	47.94

### Characteristics of lncRNAs

**Figure 2:**
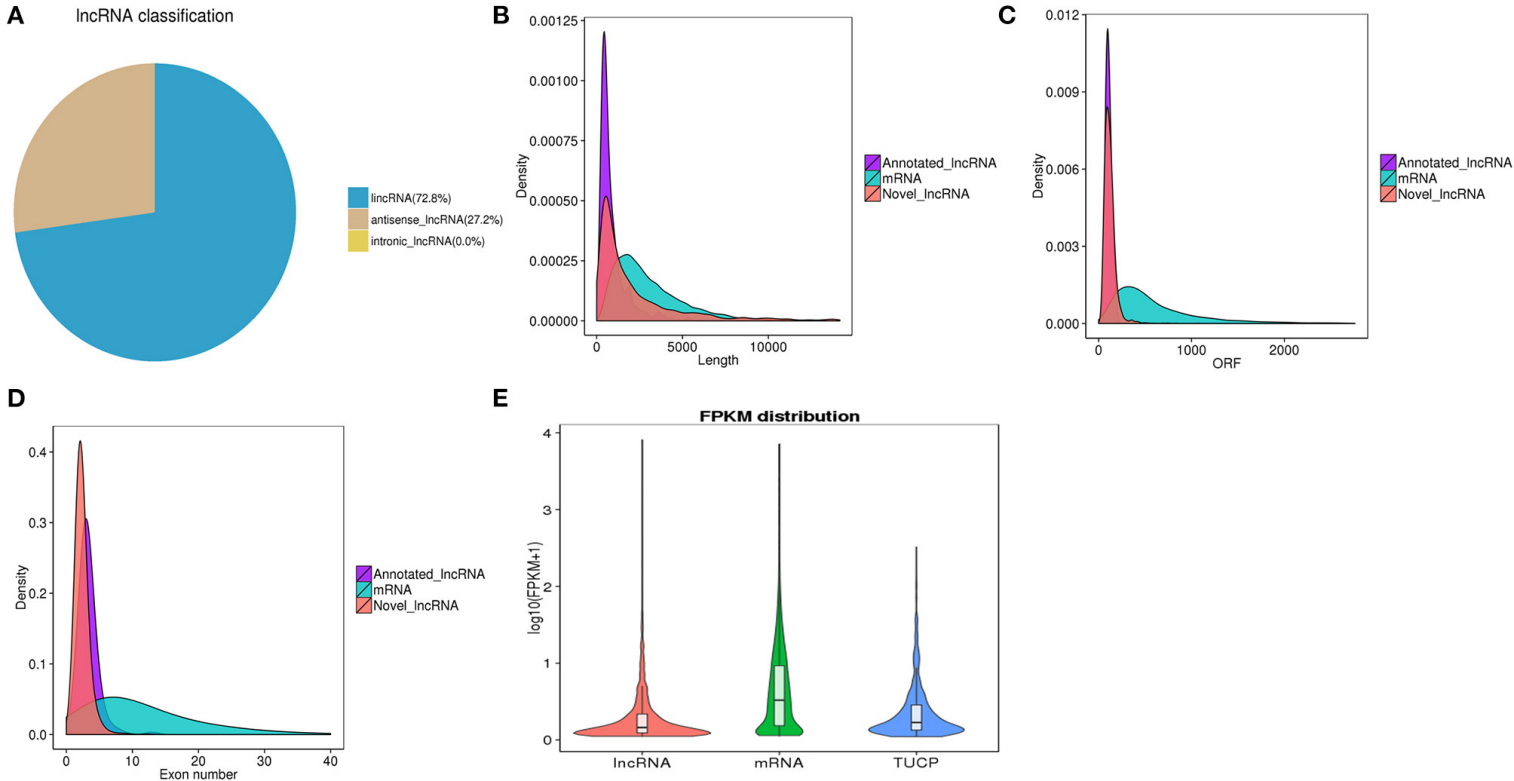
**(A)** Classification of lncRNAs in three classes. **(B)** Comparison of transcript lengths in annotated lncRNAs, mRNAs, and novel lncRNAs. **(C)** Comparison of ORF lengths in annotated lncRNAs, mRNAs, and novel lncRNAs. **(D)** Comparison of exon numbers in annotated lncRNAs, mRNAs, and novel lncRNAs. **(E)** Expression levels of lncRNAs, mRNAs, and TUCP from all samples.

LncRNAs were classified based on their genomic location, and the 4,445 lncRNAs consisted of 72.8% lincRNA (long intergenic non-coding RNA) and 27.2% antisense lncRNA but no intronic lncRNA (Figure 2A). We then compared the full length, ORF length, and exon number between the lncRNAs and mRNAs. We found that both novel and annotated lncRNAs were shorter in full length and ORF length than the mRNAs (Figures 2B,C). The ORF length of the lncRNAs ranged from 24 to 1,066 nucleotides, which was shorter than most of the mRNA ORF lengths. As shown in Figure 2D, the lncRNAs had fewer exons than the mRNAs. All the lncRNAs had 2–13 exons, while the mRNAs had a much wider distribution range of exon numbers. Otherwise, the average expression level of lncRNAs was much lower than that of mRNAs (Figure 2E).

### Different Expression Levels of lncRNAs Under *E. tarda* Infection

**Figure 3:**
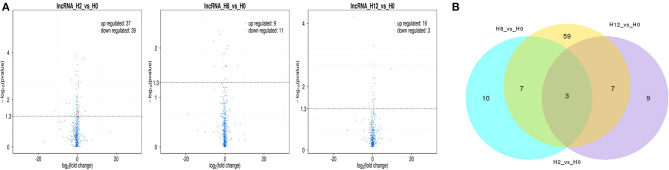
**(A)** Volcano plots of differentially expressed lncRNAs in the H2 vs. H0, H8 vs. H0, and H12 vs. H0 comparisons. The red and blue blots represent the significantly upregulated and downregulated DE-lncRNAs. **(B)** Venn diagram of the DE-lncRNAs in the three comparisons.

In comparison with H0, a total of 115 lncRNAs showed significantly different expression levels (*p*-value < 0.05), including 76, 20, and 19 DE-lncRNAs in the H2, H8, and H12 groups, respectively (Figure 3A). The most significant dfferences existed in the H2 group, in which 37 lncRNAs were significantly upregulated and 39 lncRNAs were significantly downregulated (*p*-value < 0.05). As shown in the Venn diagram, some DE-lncRNAs were differentially expressed at two or three comparisons. Moreover, despite the large number of DE-lncRNAs between the experimental and control groups, only three DE-lncRNAs (~2.6%) overlapped among three inter-group comparisons, and 14 DE-lncRNAs (~12.2%) overlapped among two inter-group comparisons (Figure 3B).

**Figure 4:**
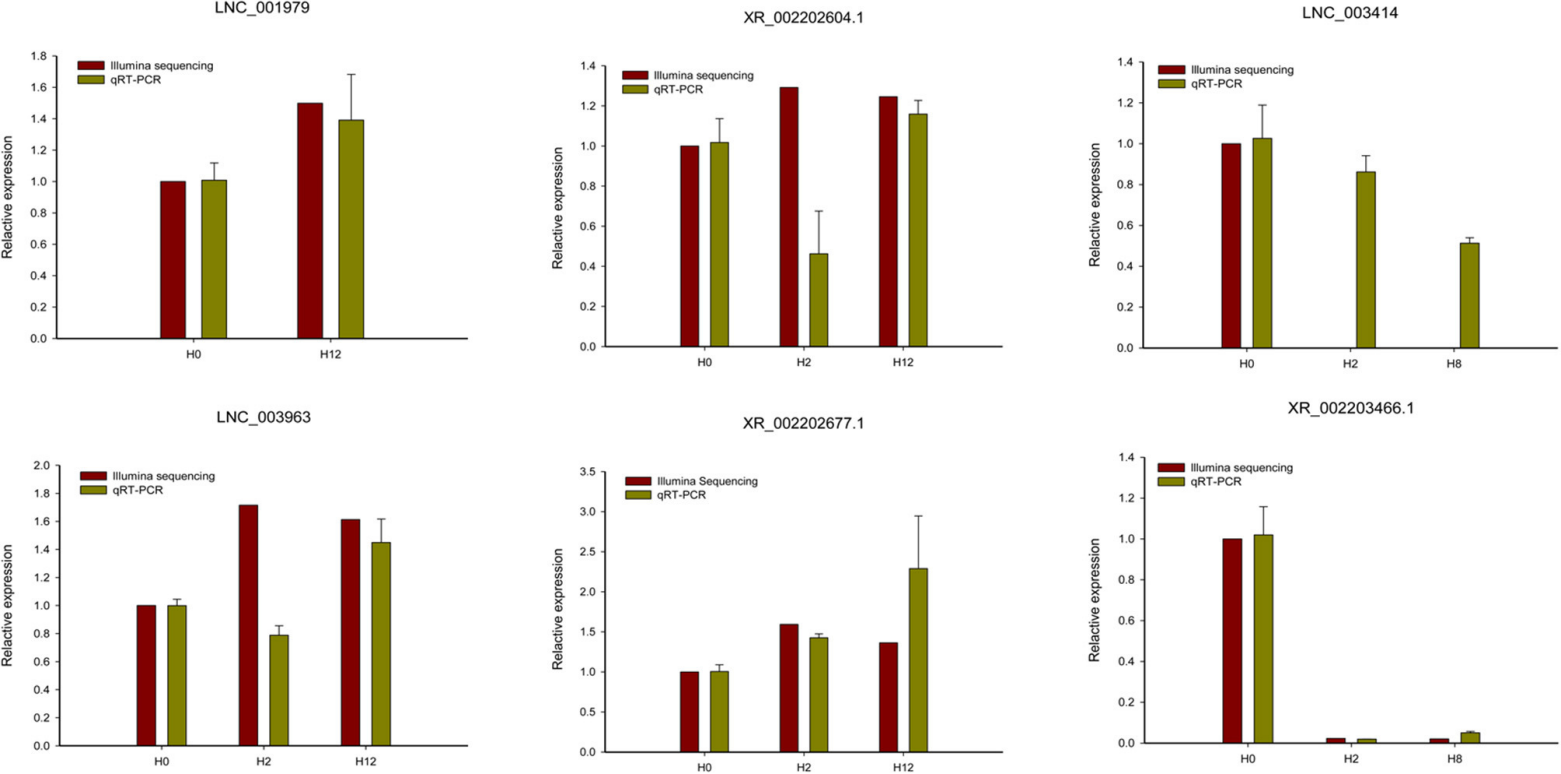
qRT-PCR analysis of six randomly selected DE-lncRNAs (LNC_001979, XR_002202604.1, LNC_003414, LNC_003963, XR_002202677.1, and XR_002203466.1). The qRT-PCR analysis results were compared with data obtained from Illumina sequencing.

To validate the RNA-seq data, six DE-lncRNAs (LNC_001979, XR_002202604.1, LNC_003414, LNC_003963, XR_002202677.1, and XR_002203466.1) were randomly selected for the qRT-PCR analysis (Figure 4). Although individual lncRNAs differed from the RNA-seq data at some time points, most of the qRT-PCR results were in high accordance with the transcriptomic results, which confirmed the reliability and accuracy of the RNA-seq data.

### Prediction of DE-lncRNA Targeted Genes

Co-localization and co-expression analyses were conducted between DE-lncRNAs and mRNAs to predict the potential lncRNA–mRNA interactions in response to bacterial infections and establish the potential roles of the lncRNAs in immunoreactions. A total of 33,531 co-location lncRNA–mRNA pairs were observed, including 3,326 lncRNAs and 18,168 mRNAs (Supplementary Table 2). We found that LNC_001129 and XR_002202865.1 exhibited the highest degree (degree = 48), followed by LNC_000228, LNC_000803, LNC_000768, LNC_000769, LNC_000774, LNC_000773, LNC_000772, LNC_000771, and LNC_000770 (degree = 45). Meanwhile, in the co-expression analysis, we observed a total of 91,501 lncRNA–mRNA pairs that contained 2,412 lncRNAs and 7,264 mRNAs (Supplementary Table 3). Of these pairs, a total of 91,221 (99.69%) were positively correlated and 280 (0.31%) were negatively correlated. We found that LNC_001467 (degree = 355) and LNC_000543 (degree = 338) indicated higher degrees. These results highlighted that lncRNAs exhibited significant expression correlations with protein-coding genes on *P. olivaceus* in response to bacterial infections.

### Function Analysis of DE-lncRNA Target Genes

To further analyze the potential function of 18,168 co-located and 7,264 co-expressed mRNAs, we analyzed their associated function using the GO enrichment and KEGG pathway analyses.

The GO enrichment analysis results are represented in Supplementary Tables 4, 5 for co-located and co-expressed mRNAs, respectively. In this study, numerous immune-related processes were enriched in biological processes, such as the intracellular transport of viral proteins in host cells (GO:0019060), regulation of viral protein levels in host cells by viruses (GO:0046719), bacteriocin immunity (GO:0030153), humoral immune response (GO:0006959), innate immune response (GO:0045087), evasion or tolerance of the host defense response (GO:0030682), avoidance of host defenses (GO:0044413).

**Figure 5:**
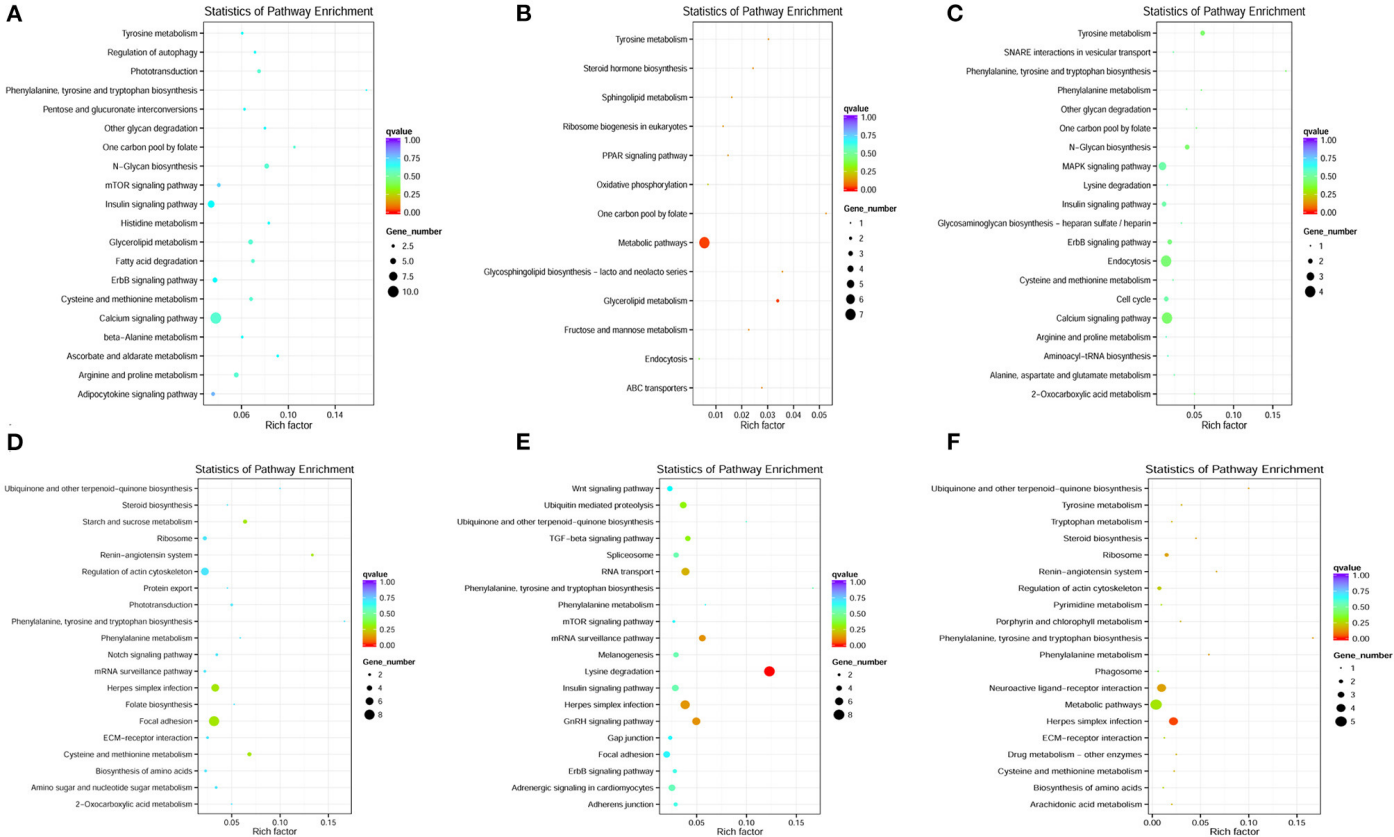
KEGG pathway enrichment of co-located and co-expressed mRNAs. **(A–C)** The most significantly enriched top 20 pathways of co-located mRNAs in the H2 vs. H0, H8 vs. H0, and H12 vs. H0 comparisons, respectively. **(D–F)** The most significantly enriched top 20 pathways of co-expressed mRNAs in the H2 vs. H0, H8 vs. H0, and H12 vs. H0 comparisons, respectively.

Otherwise, the most significantly enriched top 20 pathways were selected to represent the KEGG pathway enrichment results. As shown in Figure 5, the KEGG pathway enrichment results shown that the target genes of DE-lncRNAs exhibited a strong correlation with immune-related signaling pathways, including the regulation of autophagy, the PPAR signaling pathway, endocytosis, the MAPK signaling pathway, the Notch signaling pathway, herpes simplex infections, ECM–receptor interactions, and phagosomes. This suggested that lncRNAs may play essential roles in modulating mRNA expression levels and subsequently trigger downstream immune signaling pathways to regulate the immune response to pathogen infections in fish.

### Bioinformatics Analysis of lncRNA–miRNA–mRNA Networks

**Figure 6:**
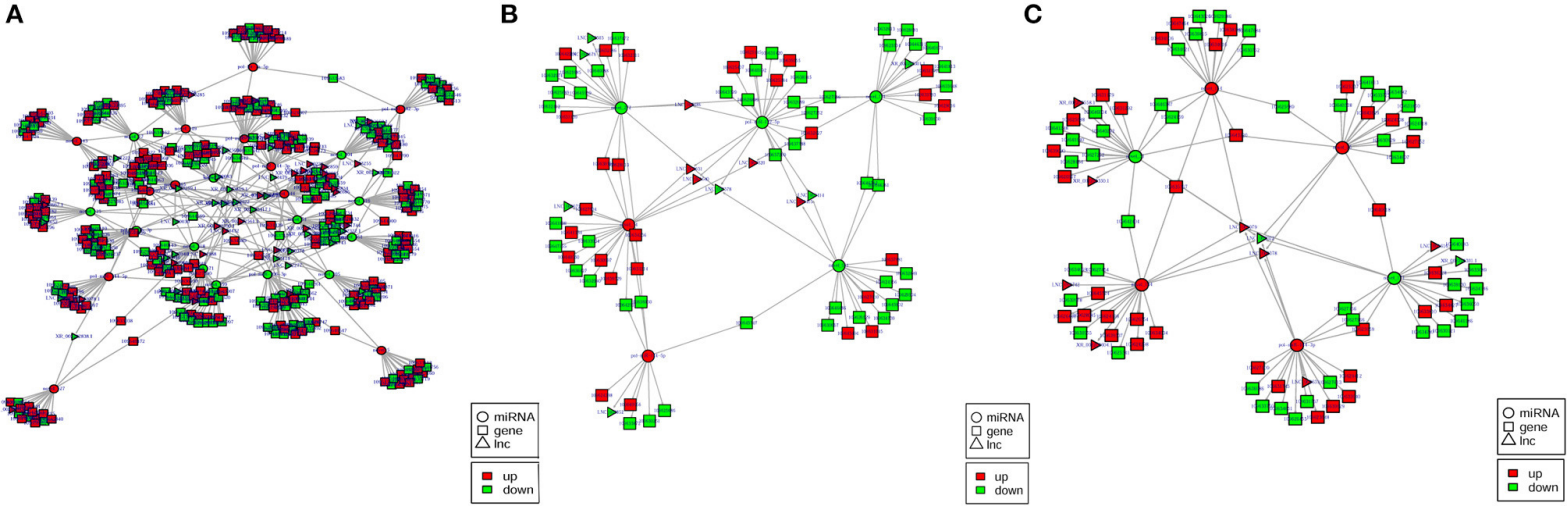
**(A–C)** The predicted lncRNA–miRNA–mRNA networks in the H2 vs. H0, H8 vs. H0, and H12 vs. H0 comparisons, respectively. LncRNA, miRNA, and mRNA are represented by triangles, circles, and squares, respectively. The red color represents upregulated, and the green color represents downregulated.

To better comprehend the role of differentially expressed lncRNAs, lncRNA–miRNA–mRNA ceRNA triple regulatory networks were constructed. The lncRNA–miRNA–mRNA networks were generated using a combination of lncRNA–miRNA pairs and miRNA–mRNA pairs, which were predicted using the MiRanda software based on their differentially expressed results (Figure 6). This network contained 169 lncRNA–miRNA pairs and 3,682 miRNA–mRNA pairs, including 64 lncRNAs, 31 miRNAs, and 1,766 mRNAs (Supplementary Table 6).

Among the 169 lncRNA–miRNA pairs, a few circRNA-miRNA pairs existed in multiple comparison groups; for example, XR_002202301.1-novel_171 existed in all three comparisons, LNC_003414-novel_561, LNC_003415-novel_561, and LNC_000378-novel_561 existed in both H2 vs. H0 and H8 vs. H0 comparisons, XR_002202604.1-novel_144, LNC_002022-novel_144, LNC_000378-novel_144, LNC_002022-novel_171, LNC_002022-novel_51, XR_002202350.1-novel_51, LNC_002022-pol-miR-144-3p, LNC_000378-pol-miR-144-3p, and LNC_003631-pol-miR-144-3p existed in both H2 vs. H0 and H12 vs. H0 comparisons. Among the 3,682 miRNA–mRNA pairs, 179 miRNA–mRNA pairs existed in multiple comparison groups; for example, novel_171-109627566, novel_171-109625534, novel_171-109646742, novel_171-109646311, novel_171-109644261, novel_171-109632613, novel_171-109639130, novel_171-109641813, novel_171-109624060, novel_171-109639395, novel_171-109634146, and novel_171-109631850 pairs existed in all of the three comparisons.

### Potential LNC_001979-Novel_171-mRNA (Potusc2 and Podad1) ceRNA Network

**Figure 7:**
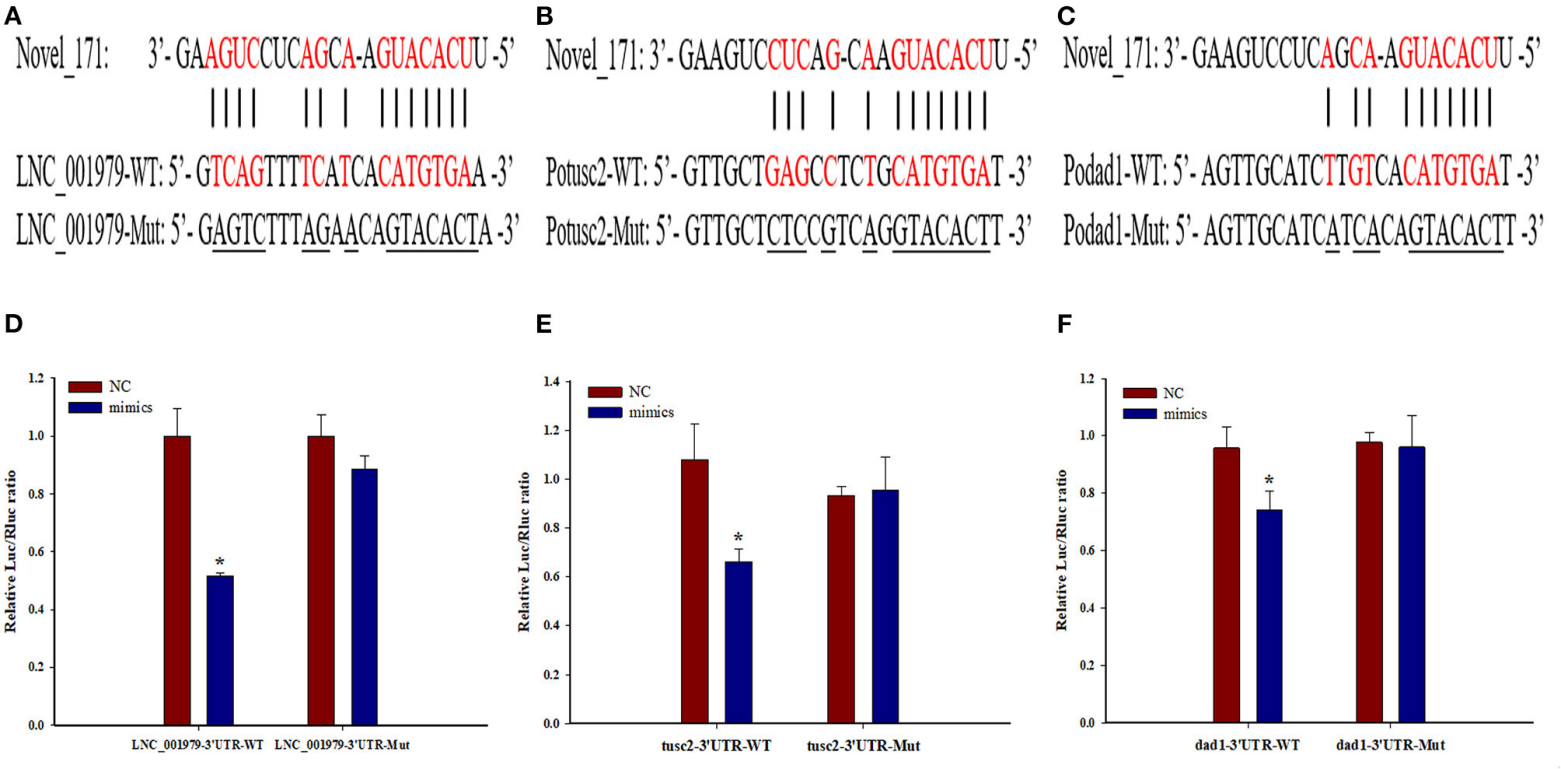
The potential LNC_001979-novel_171-mRNA (Potusc2 and Podad1) ceRNA network. Sequence alignment of novel_171 and its binding sites in the LNC_001979 **(A)**, Potusc2 **(B)**, and Podad1 **(C)**. HEK293 cells were transfected with wild-type or mutant LNC_001979 **(D)**, Potusc2 **(E)**, and Podad1 **(F)**, together with novel_171 mimics, or NC. The asterisks (*) indicate significant differences (*P* < 0.05) between the NC and mimics group.

Bioinformatics analyses revealed that LNC_001979, Potusc2, and Podad1 harbor a common standard target sequence for novel_171 (Figures 7A–C). To investigate whether LNC_001979, Potusc2, and Podad1 are direct targets of novel_171, a dual-luciferase reporter assay was performed. As shown in Figures 7D–F, novel_171 mimics the markedly decreased luciferase activity of cells transfected with pmirGLO-LNC_001979-WT, pmirGLO-Potusc2-WT, or pmirGLO-Podad1-WT, but no effect on luciferase activity was observed in cells transfected with pmirGLO-LNC_001979-Mut, pmirGLO-Potusc2-Mut, or pmirGLO-Podad1-Mut. The novel_171 negative control was also subjected to HEK293 cells for luciferase activity, there was no effect on luciferase activity in cells transfected with wild or mutant plasmids. These results revealed that novel_171 can directly target LNC_001979, Potusc2, and Podad1.

## Discussion

### Transcriptome Assembly

Recently, an increasing number of lncRNAs have been identified and characterized from different immune-related tissues of teleosts using high-throughput sequencing, some of which have been proven to play important roles in immune responses against pathogen infections (26). In this study, the Illumina platform was used to investigate the lncRNA profile of olive flounder. More complete and unbiased transcriptome datasets were developed, which will help elucidating the function of lncRNAs related to immune responses under pathogen infections in teleosts.

In this study, each library produced more than 83 million raw reads. After filtering the low-quality reads, a total of 287,208,722, 294,346,456, 254,332,520, and 329,139,946 clean reads were acquired from the H0, H2, H8, and H12 group, respectively, which accounted for more than 93% of the raw reads. Furthermore, at least 84% of the clean reads from each library were mapped on to the genome of the olive flounder, which is higher than what has been previously reported. Previous reports have shown that the mapping rates were only 67.32, 74, and 79.13% (18, 27), which are relatively low. It has been speculated that the low mapping rates of olive flounders may be due to their imperfect reference genome (18). In conclusion, we obtained good clean reads and mapping rates, which is of great significant for further studies on screening and verifying the roles of lncRNAs.

A total of 4,445 putative lncRNAs were identified, including 3,975 novel lncRNAs and 470 annotated lncRNAs, which tremendously enriched the pool of lncRNAs in fish intestine. The intestine, which is one of the main mucosa-associated lymphoid tissues in teleosts (28), was likely the main route of *E. tarda* entry (29, 30). Despite evidence for the immune functions of teleost intestines, no study on lncRNAs has exclusively focused on intestinal tissue; most of the studies have just been conducted on the head kidney, spleen, and liver. Otherwise, we found that olive flounder lncRNAs share several more common characteristics of lncRNAs than mRNAs, including shorter full lengths and ORF lengths, fewer exons, sequence length, lower expression levels, and sequence conservation (31).

### DE-lncRNAs and Annotation of Target Genes

Recently, a growing body of literature identified that lncRNAs acting as positive or negative regulators in immunity against bacterial infection (32). On the one hand, the host lncRNAs play important roles in protecting host from pathogen invasion by regulating immune-related genes at epigenetic, transcriptional, and post-transcriptional levels (32). In the epigenetic modification, lncRNAs regulate DNA methylation and histone modification to change the state of chromatin, thereby leading to transcriptional activation or silencing (32). At the transcriptional level, lncRNAs can directly affect the transcription of downstream genes by physically interacting with transcription factors, structural proteins, and RNA binding proteins (33). At the post-transcriptional level, lncRNAs affect gene expression by regulating translation efficiency, mRNA stability, and splicing (5). On the other hand, bacteria can manipulate the host signaling pathways by regulating the host lncRNAs to escape immune clearance (32), for example, two lncRNAs, SSR42 and RNA III, participated in alpha-toxin production and *Staphylococcus aureus* hemolysis induced by antibiotics (34, 35).

In the last few years, the modulation of lncRNAs has been described in teleosts after infected with viruses, bacteria, or parasites (3, 6–13). In order to identify the lncRNAs that are involved in the defense of olive flounder against *E. tarda* infections, the expression levels of lncRNAs were calculated and the DE-lncRNAs between the experimental and control groups were filtered. A total of 115 DE-lncRNAs were identified, which consisted of 76, 20, and 19 DE-lncRNAs in the H2 vs. H0, H8 vs. H0, and H12 vs. H0 comparisons, respectively. We discovered that lncRNAs were expressed in a time-dependent manner post *E. tarda* infection, which indicated that DE-lncRNAs may exert a different regulatory role in the intestine of olive flounder. Moreover, our results indicate that lncRNAs might mainly participate in the early stage of host immune responses. Similarly, in the European sea bass, the number of DE-lncRNAs substantially reduced as time progressed; for example, a total of 204 and 931 lncRNAs were significantly modulated in the head kidney and brain of European sea bass 24 h after nodavirus infection, but only 93 lncRNAs and 342 lncRNAs were significantly modulated at 72 h (13). In the last few years, DE-lncRNAs have been linked to the fluctuations in typical immune-related genes. Furthermore, the described modulatory functions of DE-lncRNAs have mainly been related to their impact on the co-located and co-expressed protein-coding target genes. In this study, a total of 18,168 co-located and 7,264 co-expressed target genes were discovered, which were then annotated with GO and KEGG function databases.

The GO analysis identified that the target genes of DE-lncRNAs participated in diverse biological processes under the infectious agent. Furthermore, several target genes of DE-lncRNAs were enriched in several immune-related processes, which suggested that lncRNAs serve as intermediaries and play a significant role in regulating immune responses. A similar discovery was reported on the European sea bass, in which the GO analysis showed that numerous biological process terms directly involved in immunity were found to be enriched at 24 h post-challenge (13).

The KEGG analysis enables a better understanding of the complex network in regulatory mechanisms (17). Our results identified that the target genes of DE-lncRNAs were strongly enriched in immune-related signaling pathways, including the regulation of autophagy, the PPAR signaling pathway, endocytosis, the MAPK signaling pathway, the Notch signaling pathway, herpes simplex infection, ECM-receptor interactions, and phagosomes. Immune-related signaling pathways that are enriched by lncRNA target genes have been found in other teleosts. For example, the KEGG analysis of zebrafish showed a large number of processes linked to viral infections, such as endocytosis, the MAPK signaling pathway, herpes simplex infection, the Toll-like receptor signaling pathway, the RIG-I-like receptor signaling pathway, and the NOD-like receptor signaling pathway (12). Paneru et al. (6) reported that a total of 290 neighboring gene of DE-lncRNAs in rainbow trout had hits to KEGG pathways, in which 51 different genes were related to immunity pathways, including chemokine signaling, platelet activation, complement system, TNF signaling, T-cell receptor signaling, Fc gamma R-mediated phagocytosis, Toll-like receptor signaling, phagosomes, cytokine-cytokine receptor interactions, NOD-like receptor signaling, leukocyte trans-endothelial migration, and others. In addition, 49 different genes were involved in microbial infection processes and 28 different genes were common in both sets of these pathways. Otherwise, lncRNAs stimulated the TLR signaling pathway to elicit host antiviral responses in yellow croaker post *Vibrio anguillarum* infection (36). Therefore, lncRNAs may play central and diverse roles in controlling host immune responses against pathogen infections via triggering downstream immune signaling pathways.

### lncRNA–miRNA–mRNA Networks in Olive Flounder

An increasing number of studies have confirmed that lncRNAs can act as targets of miRNAs and then suppress the interaction between miRNAs and coding genes (6, 13). In recent years, ceRNA regulatory networks have been widely investigated in types of diseases (11, 37). Moreover, Chu et al. (27, 38) confirmed the hypothesis that ceRNA regulatory networks also exist in teleosts. In this study, the lncRNA–miRNA–mRNA networks were constructed by using a combination of lncRNA–miRNA and miRNA–mRNA pairs. KEGG analysis revealed that mRNAs in the lncRNA–miRNA–mRNA networks were significantly (*p* < 0.05) enriched in Herpes simplex infection. A total of 32 mRNAs were involved in herpes simplex infection signaling pathway, including 109,626,283, 109,623,691, 109,627,599, 109,644,197, 109,647,155, 109,641,940, 109,625,845, 109,637,327, 109,624,406, 109,633,274, 109,643,961, 109,636,767, 109,628,267, 109,643,520, 109,639,858, 109,644,261, 109,625,570, 109,634,833, 109,633,363, 109,643,253, 109,643,252, 109,637,639, 109,626,354, 109,631,327, 109,642,261, 109,641,879, 109,629,246, 109,641,908, 109,629,344, 109,646,115, 109,645,569 and 109,633,948. Herpes simplex virus (HSV) is a common human pathogen, which initially infects orofacial mucosal surfaces and replicates in epithelial cells at these sites, causing clinically overt disease characterized by vesicular lesions (39). On one hand, HSV invasion is normally followed by activation of both the innate and adaptive immune systems (40). On the other hand, HSV develops different mechanisms, including inhibition of autophagy and apoptosis to avoid the immune system and maintain itself in latency (40). This research revealed that Herpes simplex infection signaling pathway is also important in the regulation of *E. tarda* infection, and the constructed lncRNA–miRNA–mRNA networks shed new light on understanding the interplay of *E. tarda* infection and the intestinal mucosal immune responses of *P. olivaceus*.

Recently, it has been proven that lncRNAs serve as novel regulators for innate antiviral responses in teleost fish. The lncRNA MARL functions as a ceRNA for miR-122 to control the abundance of mitochondrial antiviral signaling proteins (MAVS), thereby inhibiting *Siniperca chuatsi rhabdovirus* (SCRV) replication and promoting antiviral responses (38). In addition, the lncRNA AANCR functions as a ceRNA for miR-210 to control the protein abundance of MITA, thereby inhibiting SCRV replication and promoting antiviral responses (27). To the best of our knowledge, the present study is the first to identify two potential ceRNA regulatory networks, LNC_001979-novel_171-Potusc2 and LNC_001979-novel_171-Podad1, from the intestine of olive flounder. Both the dad1 (defender against cell death 1) and tusc2 (tumor suppressor candidate 2) encode multifunctional protein that play an important role in regulating a wide range of cellular processes. The dad1, highly conserved from yeast to mammals, act as regulatory protein to inhibit the programmed cell death which restricts the pathogens multiply and spreading in host tissue by killing pathogen-infected cells (41, 42). Several plant dad1 orthologs have been proved to play a critical role in defense against Phytophthora pathogens and might participate in the ER stress signaling pathway (43). Besides, dad1 is required for proper processing of N-linked glycoproteins and for certain cell survival in the mouse (44), and functional loss of dad1 in Drosophila would lead to a reduction of tissue growth due to increased apoptosis and lack of cell proliferation (45). The tusc2, a known tumor suppressor gene, is downregulated in non-small cell lung carcinomas, small cell lung carcinomas, mesothelioma, esophageal carcinoma, thyroid carcinoma, glioblastoma and sarcomas (46). The TUSC2 protein plays an important role in regulating a wide range of cellular processes, such as cell cycle arrest and apoptosis, in modulating the function of several kinases and affecting gene expression (47, 48). These two potential ceRNA regulatory networks were constructed based on the target prediction of novel_171, dual-luciferase reporter assays, and their relative expression levels during *E. tarda* infection. However, further studies should be conducted to confirm these two ceRNA regulatory networks and elucidate their roles in the immune responses of olive flounder intestine.

## Data Availability Statement

The original contributions presented in the study are included in the article/Supplementary Material, further inquiries can be directed to the corresponding author/s.

## Ethics Statement

The animal study was reviewed and approved by Qingdao Agricultural University.

## Author Contributions

YX: conduct lncRNA sequencing and wrote this paper. YL: analyze lncRNA data. XL: raise the experimental fish. LS: conduct the bacteria challenge experiment. SZ: revised the manuscript. CL: conceived and designed the research, and revised the manuscript. All authors: contributed to the article and approved the submitted version.

## Conflict of Interest

The authors declare that the research was conducted in the absence of any commercial or financial relationships that could be construed as a potential conflict of interest.
